# Fish Food in the Deep Sea: Revisiting the Role of Large Food-Falls

**DOI:** 10.1371/journal.pone.0096016

**Published:** 2014-05-07

**Authors:** Nicholas D. Higgs, Andrew R. Gates, Daniel O. B. Jones

**Affiliations:** 1 Marine Institute, Plymouth University, Drake Circus, Plymouth, United Kingdom; 2 SERPENT Project, National Oceanography Centre, Southampton, United Kingdom; Dauphin Island Sea Lab, United States of America

## Abstract

The carcasses of large pelagic vertebrates that sink to the seafloor represent a bounty of food to the deep-sea benthos, but natural food-falls have been rarely observed. Here were report on the first observations of three large ‘fish-falls’ on the deep-sea floor: a whale shark (*Rhincodon typus*) and three mobulid rays (genus *Mobula*). These observations come from industrial remotely operated vehicle video surveys of the seafloor on the Angola continental margin. The carcasses supported moderate communities of scavenging fish (up to 50 individuals per carcass), mostly from the family Zoarcidae, which appeared to be resident on or around the remains. Based on a global dataset of scavenging rates, we estimate that the elasmobranch carcasses provided food for mobile scavengers over extended time periods from weeks to months. No evidence of whale-fall type communities was observed on or around the carcasses, with the exception of putative sulphide-oxidising bacterial mats that outlined one of the mobulid carcasses. Using best estimates of carcass mass, we calculate that the carcasses reported here represent an average supply of carbon to the local seafloor of 0.4 mg m^−2^d^−1^, equivalent to ∼4% of the normal particulate organic carbon flux. Rapid flux of high-quality labile organic carbon in fish carcasses increases the transfer efficiency of the biological pump of carbon from the surface oceans to the deep sea. We postulate that these food-falls are the result of a local concentration of large marine vertebrates, linked to the high surface primary productivity in the study area.

## Introduction

In the absence of sunlight, most animals in the deep ocean (below 200 m) are reliant on detritus from the surface waters as their primary source of food. This is mainly composed of dead plankton and fecal pellets produced by zooplankton, which are exported to the deep seafloor as fine particles of ‘marine snow’. Particulate organic carbon (POC) export to the deep-sea decreases exponentially with depth and is believed to play a key role in structuring deep-sea communities[1]–[3]. Temporal fluctuations in the quantity and quality of POC can have marked effects on the benthic community below, and some animals appear to be specially adapted to respond to these changes[4].

While most detritus reaches the seafloor as millimetre sized particles of marine snow, the remains of large plants, algae and animals arrive as bulk parcels that create areas of intense organic enrichment. Early investigation of this phenomenon looked at the utilisation of wood and other plant remains in the deep-sea [5]–[7], while baited camera traps revealed a host of scavengers that consumed animal carcasses [8], [9]. Additionally, chance photographs of intact mammal carcasses and skeletons on the deep-sea floor were also reported [10]–[12], prompting discussion of the role that food-falls play in deep-sea food chains [13]. Specifically, it was doubted that food-falls could be frequent enough to support apparent specialist scavengers [14].

These discussions were brought to the fore with the serendipitous discovery of an intact whale skeleton at bathyal depths off California [15]. Intriguingly, the skeleton hosted chemoautotrophic fauna similar to those seen at hydrothermal vents, thriving off hydrogen sulphide generated by the anaerobic decomposition of skeletal lipids [16]. This finding showed that the very largest food-falls may play more ecologically significant roles than simply feeding scavengers. Subsequent studies of both natural and experimentally implanted whale carcasses (whale-falls) have provided evidence that these habitats go through several ecological stages in which different trophic guilds dominate [17], [18]. This ecological succession is responsible for the comparatively high species diversity at found whale-fall habitats [19], including specialists such as the bone-eating *Osedax* worms [20] and bone-eating snails *Rubyspira* [21]. Thus, whale-falls may play a significant role in maintaining biodiversity over ecological and evolutionary time scales by increasing the range of ecological niche space [22], [23].

The enhanced diversity associated with whale-fall habitats has been attributed to their large size, high lipid content of the bones, and their multi-decadal persistence on the seafloor [24]–[26], all of which are probably interrelated [27]. There has been much speculation about the ability of non-mammalian food-falls to host whale-fall type communities [28]–[30] and the carcass size required to attract and sustain whale-fall communities [31]–[33], but studies on the fate of vertebrate remains at bathyal depths have been restricted to either small porpoise and dolphin carcasses [34], [35] or large whale carcasses e.g. [27], [36]. Here we report the on chance discovery of several large ‘fish-falls’ comprised of a whale shark carcass and three mobulid ray carcasses from the deep seafloor off Angola. We describe the associated fauna and discuss the role of these large food-falls on deep-sea ecosystems.

## Materials and Methods

**Figure 1:**
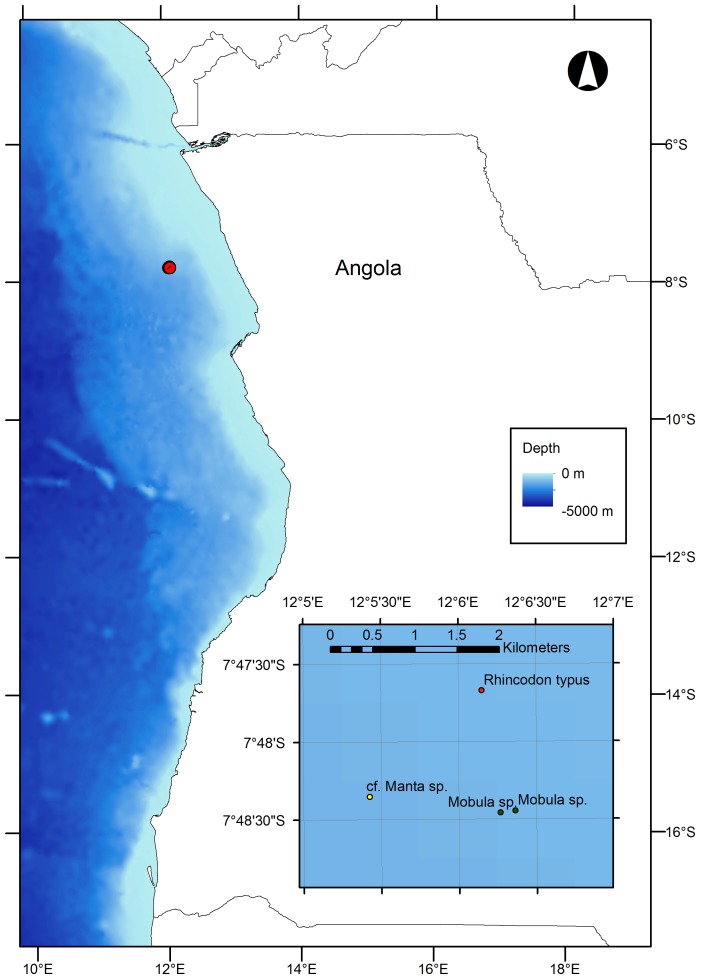
Map showing the locations of elasmobranch carcasses (inset) observed on the Angola continental margin.

Standard definition video of chance encounters with large elasmobranch carcasses observed during Subsea 7 *Hercules* remotely operated vehicle (ROV) operations from the vessel M. V. *Bourbon Oceanteam 101* were analysed. Four clips collected between 22^nd^ June 2008 and 14^th^ May 2010, ranging from 29 seconds to 4 minutes 58 seconds in length were available for analysis. Full details of the video are shown in Table 1. The video data were collected as part of seafloor surveys of seafloor structures undertaken by BP Angola and partners in license block 18 offshore of Angola (Figure 1). The data were made available for analysis via the SERPENT Project (www.serpentproject.com).

**Table 1:** Details of carcasses encountered on the Angola continental margin.

ID#	Species	Date	Clip length	Latitude (°)	Longitude (°)	Depth (m)	Estimated	Estimated Minimum
		Discovered	(min:sec)				Mass (kg)	Time on Bottom (d)
	*Rhincodon typus*	22/06/08	00:29	−7.790695	12.102554	1210	3600	60
1	*Mobula* sp.	03/05/09	04:58	−7.803794	12.104504	1233	100	7−14
2	cf. *Manta* sp.	18/01/10	00:58	−7.802029	12.090473	1235	>200	14
3	*Mobula* sp.	14/05/10	01:46	−7.803577	12.106118	1233	100	7−14

The opportunistic nature of the data collection resulted in several limitations. No faunal samples were collected, so all taxonomic identifications are tentative (Table 2), based on consultation with a selection of taxonomic experts. There is no available indication of the sampling effort required to obtain these four observations and there was no inherent sampling design in the observations. The industrial class ROVs that were used to undertake the surveys were not fitted with parallel lasers for determining scale, making it difficult to attain accurate size measurements of objects in the videos. As a result the data reported here is primarily qualitative, using best estimates of size where necessary, based on previously published data.

**Table 2:** Fauna observed at elasmobranch carcasses.

Carcass	Taxon observed	Abundance
Whale shark	*Pachycara crassiceps*	18
Mobulid carcass 1	Asteroidea	1
	*Myxine ios*	1
	*Coryphaenoides* sp.	1
	*Bathyraja* sp.	1
	*Synaphobranchus kaupii*	2
	*Pachycara* sp.	20
Mobulid carcass 2	*Pachycara* sp.	54
Mobulid carcass 3	*Lycodes terranovae*	13

Quantitative values for comparison with previous studies were calculated by combining the occurrence data from the video with best estimates from the literature. Values for carcass size were used to estimate the time that each carcass had been on the seafloor, based on previously recorded rates of scavenging in the deep sea. The mass and time values for each carcass were then integrated over the entire area bounding their occurrence (convex hull) to obtain an estimate of the mass flux to that area of seabed. Mass flux was converted to carbon flux according to empirically determined conversion factors reported in the literature. These calculations are sensitive to changes in the area that the carcass mass is integrated over and we chose the convex hull since it requires the fewest assumptions. We consider this a conservative estimate since much of this area was not surveyed and may have contained further carcasses.

## Results and Discussion

During routine seabed surveys over the course of two years, the carcass of a whale shark and three mobulid rays were found by chance on the seafloor at bathyal depths on the Angola continental margin (Figure 1). It is extremely rare to encounter natural food-falls; in five decades of deep-sea photography and exploration only nine vertebrate carcasses have ever been documented[10]–[12], [15], [27], [37]–[40]. To find four in such close proximity is unprecedented, suggesting that large food-falls are common in the region. The cause of death of the animals identified here is unknown and most carcasses appear to have arrived at the seabed intact (see below). There is no targeted fishery for whale sharks and mobulid rays off Angola, but ship strikes and accidental entanglement are common sources of anthropogenic mortality [41], [42]. Natural mortality is usually the result of opportunistic attacks by sharks and killer whales [43]–[45].

### Whale shark carcass

**Figure 2:**
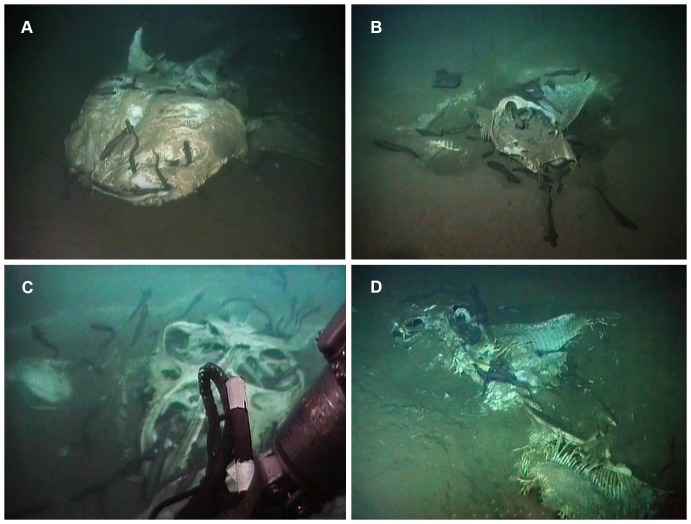
Still images showing each of the observed carcasses. A Whale shark (*Rhincodon typus*); B Mobulid carcass 1; C Mobulid carcass 2; D Mobulid Carcass 3. Images have been enhanced. Originals and details of enhancements are available in Figure S1.

Whale sharks (*Rhincodon typus*) have only recently been documented in oceanic waters off of Angola, and appear to be more common in water depths over 1,000 m in this region [46]. This affinity for deep-water suggests that whale shark carcasses may be a common form of food-fall for deep-sea scavengers in this area. The remnants of a whale shark were found at a depth of 1210 m, resting dorsal-side up on the seafloor (Figure 2A). Only the anterior part of the body remained, consisting of a fleshy head, pectoral fins, pectoral girdle and a portion of the spine trailing posteriorly.

Without an accurate scale we can only speculate on the actual size of the carcass. Of the 10 records of whale sharks off Angola reported by Weir[46] eight were estimated to be in the 5–7 m length-range. One specimen observed over a prolonged period swimming next to an oil platform was estimated to be ∼15 m long [46]. Additional records of large whale-sharks off Angola come from strandings records of individuals measuring 11.5 m and 15.9 m in length [46]. If the mean length of the sighted sharks reported by Weir [46] is taken as representative we can estimate that carcass filmed here would be approximately 7.3 m long, equating to ∼3,600 kg body mass [47]. With reference to the video footage, detailed anatomical measurements of an 8.75 m long specimen [48] can provide some context of scale for features observed here: its mouth was 1.7 m wide and the distance from the snout to the origin of the pectoral fin was 2 m. The pectoral fin was 1.47 m long, measured along its outer margin and 0.67 m wide at its base.

The carcass was attended by 18 zoarcids (eelpouts), cf. *Pachycara crassiceps*, which have also been observed at baited camera traps in this area [49]. No active feeding on the carcass was observed, and most of the fish remained stationary on or near the carcass. This ‘roosting’ behavior is typical of *Pachycara* species, which have long residence times at bait [50], [51]. Although some zoarcids are thought to directly consume bait [50], benthic fauna are their main prey [52], [53] particularly small crustaceans such as amphipods[51], [54], [55]. *Pachycara* sp. have previously been observed scavenging an elasmobranch carcass, creating “long, deep groves” as they feed on amphipods that have bored into the flesh [50]. Such grooves can bee seen in the head portion of the carcass, indicating that these fish have actively fed on the carcass (Video S1). Witte [50]noted that the dorsal part of the elasmobranch carcass was the primary site of consumption by all scavengers, which might explain why the posterior part of the carcass is missing. No other fauna were observed on or around the carcass, but the camera did not allow detection of low densities of macrofauna that may have been present.

### Mobulid carcasses

As with whale sharks, the occurrence of mobulid rays (genera: *Mobula* and *Manta*) off Angola was poorly documented until recent hydrocarbon exploration facilitated dedicated surveys for marine megafauna [56] and additional recent observations at exploration drilling locations (http://archive.serpentproject.com/2148/). Sightings of mobulids observed in this region range in size between 1–3 m disc diameter, typical of *Mobula japonica/M. mobular* species complex, which was positively identified in the area [56]. This equates to a mass of 10–280 kg in weight, whereas individuals of *Manta birostris* are typically 4.5 m disc diameter, though can reach over 7 m (1,200 kg)[45], [57], [58].

#### Carcass 1

The remains of an individual *Mobula* were found 1.46 km south of the whale-shark carcass at a depth of 1233 m (Figure 2B). The skeleton was mostly intact and articulated, with its anterior-posterior axis aligned in a southeast-northwest direction. There was very little flesh remaining, and most of the right wing and head region were covered in sediment. The entire left wing was unsedimented, while the right was exposed revealing a fully articulated skeletal structure, held in place by connective tissue.

The carcass was attended by at least 20 zoarcid fish, one of which appeared to be feeding to the right of the skull (Video S2). A singular hagfish, *Myxine ios* [59] was observed feeding on the left wing of the ray (time 00:14 in Video S2), before swimming off to the west of the carcass. A grenadier, possibly *Coryphaenoides marshalli* or *Coryphaenoides guentheri* (from depth distribution), was initially seen approaching the carcass from the west-southwest and abruptly changing direction towards the southeast when over the carcass. It then paused and drifted for a few seconds before heading away to the east, without directly contacting the carcass. Throughout the observation a small benthic ray (likely *Bathyraja* sp.) could be seen in the background to the northwest of the carcass. At one point it approached the carcass and skirted its posterior flank, but did not seem to come into contact with it, and then moved away again. A synaphobranchid eel, likely *Synaphobranchus kaupii*, was observed slowly swimming directly over the carcass, but again did not interact with it before approaching the ROV and swimming away. As this fish swam away another exactly like it was seen approaching the carcass, again from the northwest, and did not interact with the carcass. The only invertebrate observed was a starfish (Asteroidea), lying a short distance to the west of the carcass.

#### Carcass 2

A second mobulid carcass was found ∼1.5 km west of the first carcass at a depth of 1237 m. The skeleton was articulated and bare of flesh, but both wings were covered in sediment, with only a small portion of the left wing tip protruding above the sediment. The remainder of the skeleton rested above the sediment and was attended by at least 54 zoarcid fish. Several fish were observed actively feeding on remnants of flesh inside the skeleton, but most were inactive, exhibiting roosting behaviour as they wait to prey upon small invertebrate scavengers (Video S3).

An apparent zone of enrichment can be seen extending around the skeleton, demarcated by white mat, presumably made up of sulphide oxidising bacteria, common at whale-falls [17]. This mat represents the area of seafloor where organic matter from the carcass has become incorporated into the sediment and is being broken down anaerobically. The video footage is not of sufficient quality to permit detection of individual macrofauna but no large aggregations of chemoautotrophic clams or other fauna typical of sulphide-rich sediments could be observed in or around the zone of enrichment.

The relative size of the skeleton in relation to the zoarcids, coupled with much higher numbers of fish attending the skeleton suggests that it may be that of the larger manta ray, *Manta birostris*. Other skeletal features also suggest this may be the case, but diagnostic features at the anterior end of the carcass are not visible in the video footage, so we are unable to confirm this (M. Paig-Tran, personal communication). There is a single record of *M. birostris* off Angola, but this oceanic species is believed to be widely distributed throughout the tropics and subtropics [45].

#### Carcass 3

A third mobulid carcass (*Mobula* sp.) was found 180 m to the east of carcass 1, one year later on. The skeleton was mainly articulated, with the exception of the left wing, the remains of which appeared to be deposited at the rear of the skeleton. Thin threads of flesh were hanging on the skull and a chunk of flesh at the posterior end of the spine. The head of the carcass was pointing towards the north-northwest, indicating that it was not simply a resighting of carcass 1. Furthermore, this carcass still had flesh visible suggesting it was more recently deposited than carcass 1.

The carcass was attended by 13 zoarcid fish (cf. *Lycodes terranovae*) three of which were juveniles (Video S4). No feeding was observed and fish remained stationary until disturbed by the ROV.

### Time of deposition

It is difficult to asses the full significance of these natural food-falls without an estimate of the time that they have been on the seafloor prior to discovery. Radiochemical dating techniques have previously been used to estimate the age of several naturally occurring whale-falls [26], but without physical samples this method cannot be employed here. Another option is to compare the amount of soft tissues left on the carcasses with known scavenging rates to constrain the time that the carcasses have been on the seafloor. Deep-sea scavenging assemblages have been well documented off Southern California, where scavenging rates appears to be a logarithmic function of the carcass weight [27].

**Figure 3:**
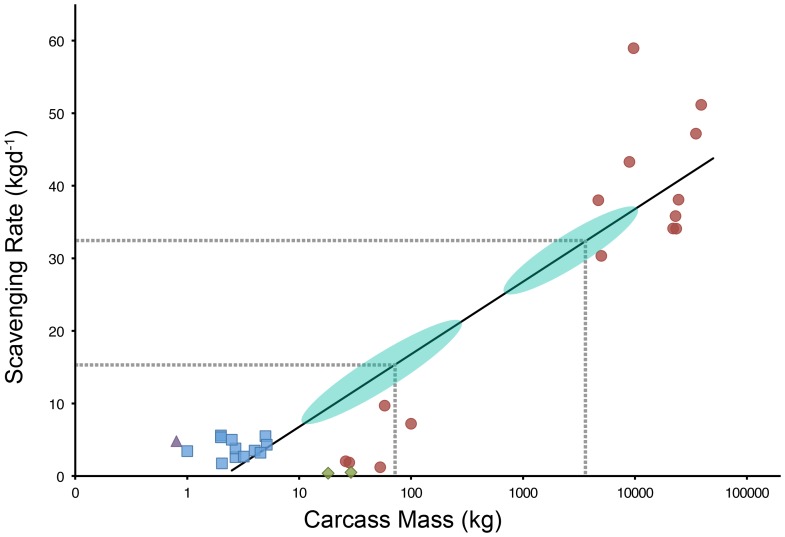
Relationship between carcass mass and the rate at which it is scavenged, based on a global dataset comprising different carcass types: •mammal; ▪ teleost; ⧫ elasmobranch; ▴ squid. A logarithmic regression (solid black line defined by the equation) *y*  =  4.345ln(*x*) − 3.222)explained a significant proportion of the variance in the relationship; R^2^ =  0.84, *F*(1,30) =  154.6, p <0.001. Data and references are presented in Dataset S1. Areas highlighted in turquoise indicate possible range in mass of the mobulid carcasses (left) and whale shark carcass (right). Corresponding dashed gray lines show best estimate for the mass of each carcass type and hence scavenging rate according to the regression equation.

Subsequent studies of food-falls (mostly whale carcasses) from several ocean basins provide data for a global analysis of carcass scavenging rates (Figure 3). The logarithmic relationship between scavenging rates and carcass size appears to be a global phenomenon, although the data are more variable than the analysis for the Southern California basins, as might be expected. According to this relationship a 3,000 kg whale shark is expected to be scavenged at a rate of ∼32 kgd^-1^, resulting in total consumption of the carcass in 3 months. Approximately 20% of the whale shark carcass was still present when observed, indicating that this carcass had been on the seafloor for over two months. For comparison a gray whale carcass, of similar size to whale shark carcass (5000 kg), had 90% of it's soft tissues removed by mobile scavengers in four months at 1220 m depth in the San Catalina Basin off California, equivalent to 37 kgd^-1^ [18]. Based on the same relationship, the smaller *Mobula* carcasses (carcasses 1 & 3) had probably been on the seafloor for at least 1-2 weeks.

There are several reasons to suggest that these values are *minimum estimates* for the amount of time that the carcasses have been present on the bottom. Very little scavenging was observed in the video footage, despite a large amount of flesh on some carcasses, showing that they were no longer being scavenged at maximal rates. Additionally, available evidence suggests that elasmobranch carrion is scavenged at much lower rates than other forms of carrion (Figure 3). When presented with elasmobranch and tuna bait on a baited camera trap, scavengers clearly preferred tuna and only consumed the elasmobranch once the tuna was gone (Jannasch 1977). Another study reported an extremely low scavenging rate of 0.38 kg/day on an elasmobranch carcass at 1900 m in the Western Arabian Sea [50]. Repeated experiments in this region using teleost fish as bait showed a 10-fold increase in scavenging rates compared to that when elasmobranch was used [51].

Depressed scavenging rates on elasmobranch carcasses may be the result of their tough, denticulate skin, making flesh difficult to access, or the flesh itself may be unpalatable. Decomposing elasmobranch flesh contains high concentrations of ammonia, related to the physiological mechanisms used in buoyancy control. Other uncharacterized chemicals that are found in rotting elasmobranch flesh (necromones) have been proven to strongly deter shark scavenging and invoke an alarm response, even among different species of elasmobranch [60]. If this phenomenon extends to deep-sea scavenging elasmobranchs, it can be assumed that the Portugese dogfish, *Centroscymnus coelolepis*, would have been deterred from scavenging the elasmobranch carcasses. This will have severely hindered utilization of the carcasses by other species, since *C. coelolepis* is the dominant scavenger off the Angola margin [49].

### Ecological role of large fish-falls

There is no evidence that the animal communities associated with the whale shark and ray carcasses had progressed beyond a scavenging stage (sensu Smith & Baco) [27]. Only mobulid carcass 2 showed signs of seabed enrichment, but the high abundance of macrofauna evident at whale-falls in the enrichment opportunist stage were not observed here. The absence of characteristic whale-fall fauna at the whale shark and mobulid carcasses may indicate that carcasses of this size and nature do not support whale-fall type communities. This is not surprising considering the nature of elasmobranch carcasses. Their flesh is primarily muscular and lacks the fatty blubber layer carried by whales. Their skeleton is unmineralised and so is prone to rapid degradation, even compared to teleost bones [61]. Indeed, fossil remains of elasmobranchs are almost exclusively restricted to teeth. Nor do their skeletons hold stores of lipid-rich bone marrow as seen in marine mammals, which may contribute to their rapid degradation [25]. At whale carcasses it is the breakdown of this lipid-rich bone marrow and blubber that generates the hydrogen sulphide and methane to support chemosynthetic fauna [16], [62], [63]. Therefore, it seems unlikely that large fish carcasses are capable of sustaining chemosynthetic fauna over ecologically significant time periods, as seen at whale-falls.

In contrast to the chemosynthetic fauna described from whale-falls, *Osedax* bone worms appear to utilise the collagen matrix of bones, not lipid-rich marrow [64] and experimental evidence shows that they are capable of living on fish bones [61]. The *Osedax* genus appears to have a wide distribution being found off California [65], Japan [66], Sweden [67] and the Southern Ocean [40], so can be expected to be found off the West Africa margin. Despite the collagen-rich food resource presented by the skeletons of the marine vertebrates, no evidence of *Osedax* could be detected on any of the carcasses. Only carcass 3 was investigated in close enough detail to detect low-density *Osedax* colonisation and no worms could be observed. In the early stages of carcass colonisation *Osedax* may not be visible in ROV surveys, but over time their abundance increases to the point at which they densely cover bone surfaces and become evident at the macroscale [67]. Previous studies have recorded *Osedax* colonisation on isolated carcasses within 9–10 weeks [68], [69] so it may be that the carcasses have not yet been colonised by the local *Osedax* population or juveniles may be too small to detect. It is therefore not possible to conclusively rule out the presence of *Osedax* at these carcasses, owing to the low resolution of the video footage, but observations here suggest they do not occur in the high densities reported from other whale-falls [65], [66]. The intact nature of the skeletons also seem to preclude sustained *Osedax* activity, at least on the mobulid carcasses. Perhaps *Osedax* may have been responsible for the deterioration of the posterior section of the whale shark carcass as the bones became exposed by scavengers, but no evidence of *Osedax* on the present vertebra was observed.

The observations here suggest that even the largest non-mammalian carcasses are primarily a significant food source for mobile scavengers in the deep sea and do not support further successional stages seen at whale falls. An extensive baited camera study off Angola (13 sites between 1293 m and 2453 m) showed that five species dominated the local scavenging assemblage: the Portuguese dogfish *Centroscymnus coelolepis*, the snubnosed eel *Simenchelys parasitica*, the arrow tooth eel *Synaphobranchus kaupii*, the blue hake *Antimora rostrata* and *Pachycara crassiceps* [49]. Of these, only *Synaphobranchus* and *Pachycara* were observed at the elasmobranch carcasses in this study. The reduced feeding of *C. coelolepis* at the carcasses (discussed above) would have also hindered the feeding of *S. parasitica*, which is reliant *C. coelolepis* to tear through the tough skin and expose soft flesh [49]. The total dominance of zoarcid fish at these carcasses, with occasional appearances of myxinids, macrourids and synaphobranchids is in keeping with scavenging community composition at experimental bait deployments in the Arabian Sea [51]. Numerous baited camera studies have shown that zoarcids are late-arriving scavengers, with high residence times at food-falls [34], [35], [50], [51]. Zoarcids became the dominant scavengers at a 23 kg elasmobranch carcass after ∼48 hours, (coinciding with a decrease in crustacean scavengers) and remained in high abundances until the experiment ended after 126 hours [50]. Baited camera observations have lasted only a matter hours to days, but the evidence presented here shows that zoarcid fish remain dominant at carcasses for extended time periods, in the order of months.

### Food-falls and bentho-pelagic coupling

The survey area is located just to the south of the Zaire/Congo River plume, where upwelling results in high levels of primary productivity, in the order or 100–200 mg C m^−2^d^−1^ [70], [71]. Of this ∼5–10% (9.8 mg C m^−2^d^−1^) is exported to the deep sea as particulate organic carbon (POC) [72]. However, POC flux only accounts for 25% of the carbon requirements of the deep-sea benthic community in this area [72]. This deficit is a common feature of deep-sea carbon budgets and suggests that additional sources are important in sustaining deep-sea communities [73]. Lateral carbon transfer from the continental shelf probably makes up a large proportion of the unaccounted carbon on this margin [72], but there is growing evidence that large, fast-sinking food-falls can also transfer significant amounts of carbon from the upper ocean to the deep seafloor [74]–[76].

To provide a minimum estimate of the importance of these carcasses to the biological pump some simple calculations can be made. If the estimated carbon content of the carcasses (8% wet wt.) [77], [78] is integrated over the entire area bounding their occurrence (1.23 km^2^) and over the estimated time since first and last deposition (738 days), then the average rate of carbon delivery to the seabed by the elasmobranch carcasses equates to 0.4 mg m^−2^d^−1^, i.e. 0.2% of the total surface primary production. This figure exceeds (by an order of magnitude) previous estimates of the relative importance of whale carcasses as sources of carbon. For example, Smith calculates that “it is difficult to imagine that the flux of great-whale detritus would exceed 0.3% of seafloor POC flux anywhere in the deep sea”[24], yet in this instance large elasmobranch food-falls are equivalent to 4% of the total POC flux to the seafloor. This figure is more in line with the 11–13% of POC flux, estimated to be the total contribution from carrion across all taxa to carbon input to the Santa Catalina Basin [79]. On this local scale the carcasses of planktivorous elasmobranchs appear to play a important role in the supply of organic carbon from the surface ocean to the deep seafloor. The most direct beneficiaries are deep-sea scavenger populations, which have been show to mirror fluctuations in the abundance of fish in surface waters [80].

Whale sharks and mobulid rays feed directly on patchy dense aggregations of zooplankton, which means that their distributions are closely linked to environmental determinants of food availability; more so than other large marine animals that feed at higher trophic levels [81]. Sea-surface temperatures have been found to be the best correlate of whale shark sightings in oceanic waters, with 90% of sightings in the Indian Ocean occurring between 26–30°C [82]. Sea-surface temperatures off northern Angola, especially in spring and summer, closely match this optimal temperature envelope [71] and sightings of whale sharks appear to corroborate the association [46]. Mobulids show a preference for slightly cooler waters in the range of 20–26°C [45], which is more characteristic of autumn and winter sea surface temperatures off Angola [71]. This area of the Angola margin also has a particularly rich cetacean fauna with high relative abundances of sperm whales and humpback whales [83] and sightings of the most massive bony fish, the ocean sunfish (*Mola mola*), are common [56], probably related to their diet of zooplankton [84]. We therefore suggest that this region is a ‘hotspot’ for planktivorous megafauna, created by a combination of high surface primary productivity and optimal temperature ranges for poikilothermic plankton feeders.

Pelagic communities that have a high proportion of large planktivorous megafauna are expected to have an increased flux of carbon from surface to deep waters through two interrelated mechanisms. Firstly, surface primary production can support a higher total biomass of large animals than that of small ones because of increasing metabolic efficiencies that scale with size, i.e. less energy is lost through trophic transfer [85]. Secondly, larger animals have lower rates of predation than smaller ones, so a higher proportion of their biomass is exported to the deep sea rather than recycled in the pelagic food chain [85]. These theoretical predictions are supported by our findings, indicating an enhanced transfer efficiency of the biological pump in this area, with increased food supply to the deep-sea benthic community.

## Conclusions

In contrast to previous assumptions, food-falls of large animal carcasses can be common in parts of the deep-sea, as evidenced by the finding of four large elasmobranch carcasses over an area of just 1.48 km^2^ on the Angola continental margin. These carcasses can support scavenger communities on the deep seafloor for weeks to months at a time, but unlike larger marine mammal carcasses, they do not appear to host characteristic “whale-fall” fauna and are primarily significance for mobile scavengers. Large food-falls may be particularly frequent where oceanographic conditions create areas of high productivity, attracting planktivorous megafauna. Our results suggest that in such areas the large food-falls can account for a significant proportion of carbon export to the deep-sea, approximately 10 times larger than previous estimates for a single taxon. This increased export is expected to result in a relatively high proportion of local surface primary production reaching the deep-seafloor, supporting a more abundant community of deep-sea scavengers.

## Supporting Information

Dataset S2 Data, methodology and references used to construct Figure 3.
(DOCX)

Figure S1 Original images corresponding to those of Figure 2.
(DOCX)

Video S1 Whale-shark carcass.
(MP4)

Video S2 Mobuild carcass 1.
(MP4)

Video S3 Mobuild carcass 2.
(MP4)

Video S4 Mobuild carcass 3.
(MP4)
