# Supplementary material for: Fish Food in the Deep Sea: Revisiting the Role of Large Food-Falls
Source: PLoS One. 2014 May 7;9(5):e96016. doi: 10.1371/journal.pone.0096016 (PMC4013046; doi:10.1371/journal.pone.0096016)
Supplement: Dataset S2 — Data, methodology and references used to construct Figure 3. (DOCX) [file pone.0096016.s001.docx]

| **Region** | | **Depth (m)** | | **Carcass Species** | | **Type** | | **Carcass mass (kg)** | | **% Soft Tissues Consumed** | | **Mass Consumed (kg)** | | **Time Taken (days)** | | **Scavenging Rate (kg/day)** | | **Ref.** | |
| --- | --- | --- | --- | --- | --- | --- | --- | --- | --- | --- | --- | --- | --- | --- | --- | --- | --- | --- | --- |
| Arctic | 2644 | | *Salmo trutta, Psetta maxima, Scomber scombrus* | | Teleost | | 3 | | 100 | | 2.7 | | 1.0 | | 3 | | [1] | |  |
| Arctic | 2524 | | *Solea solea, Scomber scombrus* | | Teleost | | 3 | | 100 | | 2.7 | | 0.7 | | 4 | | [1] | |  |
| Arctic | 2377 | | *Salmo trutta, Pleuronectus platessa,* | | Teleost | | 2 | | 100 | | 2.0 | | 0.4 | | 6 | | [1] | |  |
| Arctic | 1468 | | *Salmo trutta, Solea solea, Molva molva* | | Teleost | | 5 | | 100 | | 5.0 | | 0.9 | | 6 | | [1] | |  |
| Arctic | 2504 | | *Solea solea, Belone belone* | | Teleost | | 3 | | 100 | | 2.5 | | 0.5 | | 5 | | [1] | |  |
| Arctic | 2341 | | *Solea solea, Gadus morhua* | | Teleost | | 4 | | 100 | | 4.0 | | 1.1 | | 4 | | [1] | |  |
| Arabian Sea | 3190 | | *Thunnus* sp. | | Teleost | | 3 | | 84 | | 2.7 | | 1.0 | | 3 | | [2] | |  |
| Arabian Sea | 3950 | | *Thunnus* sp. | | Teleost | | 5 | | 84 | | 4.3 | | 1.0 | | 4 | | [2] | |  |
| Arabian Sea | 4420 | | *Thunnus* sp. | | Teleost | | 5 | | 71 | | 3.2 | | 1.0 | | 3 | | [2] | |  |
| Arabian Sea | 4050 | | *Thunnus* sp. | | Teleost | | 2 | | 85 | | 1.7 | | 1.0 | | 2 | | [2] | |  |
| NE Pacific | 1310 | | *Scomber japonicas* | | Teleost | | 1 | | 100 | | 1 | | 0.3 | | 3 | | [3] | |  |
| NE Pacific | 1310 | | *Scomber japonicas* | | Teleost | | 2 | | 100 | | 2 | | 0.4 | | 5 | | [3] | |  |
| NE Pacific | 1310 | | *Scomber japonicas* | | Teleost | | 4 | | 100 | | 4 | | 0.5 | | 7 | | [3] | |  |
| S Atlantic | 1100 | | *Dissostichus eleginoides* | | Teleost | | 10 | | 100 | | 10.0 | | 3.8 | | 3 | | [4] | |  |
| S Atlantic | 900-1048 | | *Illex argentines* | | Squid | | 1 | | 100 | | 0.8 | | 0.2 | | 5 | | [4] | |  |
| Arabian Sea | 1900 | | *Charcharhinus longimani* | | Shark | | 18 | | 8 | | 1.5 | | 4.0 | | 0 | | [5] | |  |
| Arabian Sea | 4040 | | *Charcharhinus longimani* | | Shark | | 29 | | 21 | | 6.0 | | 11.0 | | 1 | | [5] | |  |
| NE Atlantic | 2555 | | *Phocoena phocoena* | | Mammal | | 26 | | 47 | | 12.3 | | 7.0 | | 2 | | [6] | |  |
| NE Atlantic | 2710 | | *Phocoena phocoena* | | Mammal | | 28 | | 100 | | 28.0 | | 15.0 | | 2 | | [6] | |  |
| NE Atlantic | 4000 | | *Phocoena phocoena* | | Mammal | | 53 | | 4 | | 2.0 | | 1.5 | | 1 | | [7] | |  |
| NE Atlantic | 4000 | | *Phocoena phocoena, Delphinus delphis* | | Mammal | | 100 | | 50 | | 50.0 | | 6.3 | | 7 | | [7] | |  |
| NE Atlantic | 4800 | | *Lagenorhynchus acutus* | | Mammal | | 58 | | 95 | | 55.1 | | 6.0 | | 10 | | [7] | |  |
| NW Pacific | 219 | | *Physeter catadon* | | Mammal | | 23000 | | 95 | | 19665 | | 549 | | 36 | | [8] | |  |
| NW Pacific | 228 | | *Physeter catadon* | | Mammal | | 39000 | | 80 | | 28080 | | 549 | | 51 | | [8] | |  |
| NW Pacific | 229 | | *Physeter catadon* | | Mammal | | 21900 | | 95 | | 18725 | | 549 | | 34 | | [8] | |  |
| NW Pacific | 254 | | *Physeter catadon* | | Mammal | | 24450 | | 95 | | 20905 | | 549 | | 38 | | [8] | |  |
| NE Pacific | 1220 | | *Eschrichtius robustus* | | Mammal | | 5000 | | 90 | | 3330 | | 122 | | 30 | | [9] | |  |
| NE Pacific | 1675 | | *Eschrichtius robustus* | | Mammal | | 35000 | | 90 | | 23310 | | 549 | | 47 | | [9] | |  |
| NE Pacific | 328 | | *Eschrichtius robustus* | | Mammal | | 4700 | | 100 | | 3478 | | 91.5 | | 38 | | [10] | |  |
| NE Pacific | 634 | | *Eschrichtius robustus* | | Mammal | | 9718 | | 100 | | 7191 | | 122.0 | | 59 | | [10] | |  |
| NE Pacific | 1018 | | *Balaenoptera musculus* | | Mammal | | 23328 | | 100 | | 14557 | | 427.0 | | 34 | | [10] | |  |
| NE Pacific | 1820 | | *Eschrichtius robustus* | | Mammal | | 8920 | | 100 | | 6600.8 | | 152.5 | | 43 | | [10] | |  |

**Supplementary Information File S1**

**Table S1:** Global dataset of carrion scavenging rates in the deep sea.

**Methodology**

For the purposes of this analysis scavenging studies were limited to those from the deep-sea below 200m.

Scavenging rates were taken directly from the references where reported (generally those ≤100kg). For those studies where not reported, rates were calculated based on information given in the references. Where carcass size was reported as it’s length, this was converted to mass based on previously published length/mass relationships^[11-12]^. The percentage of soft tissues consumed was simply calculated as the change in mass of the entire carcass over the observed time period for carcasses. For carcasses over 1,000 kg a correction for the weight of the skeleton was used based on data from the literature^[11]^. In the case of carcasses reported in reference [8] the percentage of soft tissues consumed was not directly reported, so was estimated from the written description and figures.

**References**

[1] Premke K, Klages M, Arntz WE (2006) Aggregations of Arctic deep-sea scavengers at large food falls: temporal distribution, consumption rates and population structure. Mar Ecol Prog Ser 325: 121–135.

[2] Janssen F, Treude T, Witte U (2000) Scavenger assemblages under differing trophic conditions: a case study in the deep Arabian Sea. Deep-Sea Res II 47: 2999–3026.

[3] Smith CR (1985) Food for the deep sea: utilization, dispersal, and flux of nekton falls at the Santa Catalina Basin floor. Deep-Sea Res 32: 417–442.

[4] Collins MA, Yau C, Nolan CP, Bagley PM, Priede IG (1999) Behavioural observations on the scavenging fauna of the Patagonian slope. Journal of the Marine Biological Association of the United Kingdom 79: 963–970.

[5] Witte U (1999) Consumption of large carcasses by scavenger assemblages in the deep Arabian Sea: observations by baited camera. Mar Ecol Prog Ser 183: 139–147.

[6] Kemp K, Jamieson A, Bagley P, Mcgrath H, Bailey D, et al. (2006) Consumption of large bathyal food fall, a six month study in the NE Atlantic. Mar Ecol Prog Ser 310: 65–76.

[7] Jones E, Collins M, Bagley P, Addison S, Priede I (1998) The fate of cetacean carcasses in the deep sea: observations on consumption rates and succession of scavenging species in the abyssal north-east Atlantic Ocean. P Roy Soc Lond B Bio 265: 1119–1127.

[8] Fujiwara Y, Kawato M, Yamamoto T, Yamanaka T, Sato Okoshi W, et al. (2007) Three‐year investigations into sperm whale‐fall ecosystems in Japan. Marine Ecology 28: 219–232.

[9] Smith C, Baco A, Glover AG (2002) Faunal succession on replicate deep-sea whale falls: time scales and vent-seep affinities. Cah Biol Mar 43: 293–297.

[10] Lundsten L, Schlining KL, Frasier K, Johnson SB, Kuhnz LA, et al. (2010) Time-series analysis of six whale-fall communities in Monterey Canyon, California, USA. Deep-Sea Research Part I 57: 1573–1584.

[11] Lockyer C (1976) Body weights of some species of large whales. Journal du Conseil 36: 259–273.

[12] Lockyer C, Waters T (1986) Weights and anatomical measurements of northeastern Atlantic fin (Balaenoptera physalus, Linnaeus) and sei (B. borealis, Lesson) whales. Marine Mammal Science 2: 169–185.
