# Supplementary material for: Fish Food in the Deep Sea: Revisiting the Role of Large Food-Falls
Source: PLoS One. 2014 May 7;9(5):e96016. doi: 10.1371/journal.pone.0096016 (PMC4013046; doi:10.1371/journal.pone.0096016)
Supplement: Figure S1 — Original images corresponding to those of Figure 2. (DOCX) [file pone.0096016.s002.docx]

Supporting Information File S2

**SI Figure S2:** Manipulation of original images corresponding to Figure 2, showing areas where text was removed (grey boxes).


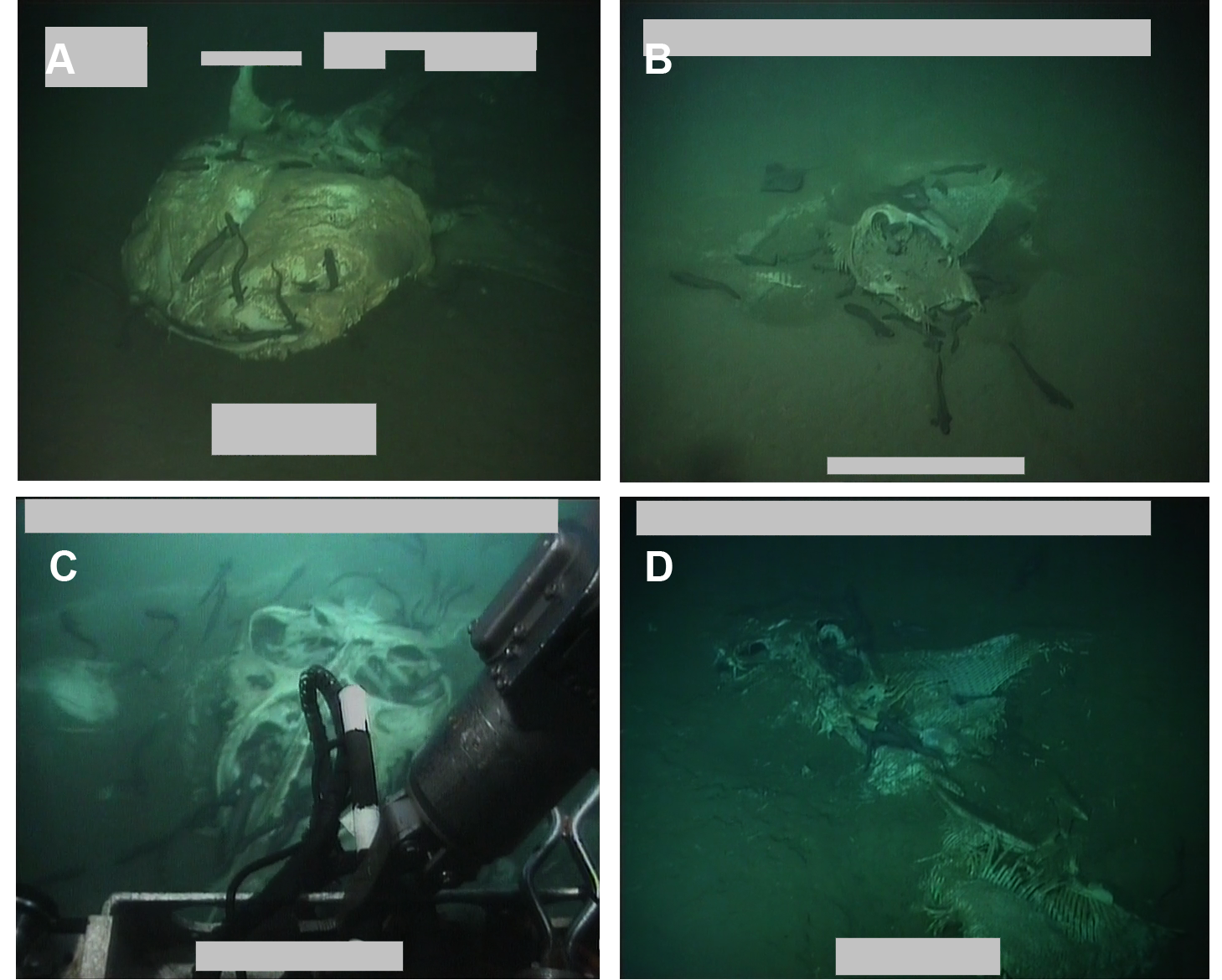


Images were cropped, resized and enhanced using Adobe Photoshop CS5.1, version 12.

Survey information burned onto images, contained in the grey boxes in Figure S2, was merged with the background image using the software’s *Healing Brush* tool.
